# Persistence of Symptoms 15 Months since COVID-19 Diagnosis: Prevalence, Risk Factors and Residual Work Ability

**DOI:** 10.3390/life13010097

**Published:** 2022-12-29

**Authors:** Donatella Sansone, Alice Tassinari, Romina Valentinotti, Dimitra Kontogiannis, Federico Ronchese, Sandro Centonze, Adele Maggiore, Luca Cegolon, Francesca Larese Filon

**Affiliations:** 1 Occupational Medicine Unit, Department of Medical, Surgical & Health Sciences, University of Trieste, 34129 Trieste, Italy; 2 Public Health Department, University Health Agency Giuliano-Isontina (ASUGI), 34128 Trieste, Italy; 3 Clinical Infectious Diseases Unit, Tor Vergata University Hospital, 00133 Rome, Italy; 4 Unit for Research on Innovation and Quality of Care, University Health Agency Giuliano-Isontina (ASUGI), 34128 Trieste, Italy

**Keywords:** SARS-CoV-2, symptoms, post-COVID-19, long-COVID-19, follow up, depression, workers, fatigue

## Abstract

**Background:** A proportion of patients’ ailments may last after recovering from acute COVID-19, with episodic and systemic symptoms of unclear etiology potentially involving different organs. **Study aim:** The aim of this study was to investigate the persistence of symptoms 15 months since COVID-19 diagnosis in patients referring to the post-COVID-19 clinic in Trieste (north-eastern Italy). **Methods:** Two-hundred-forty-seven patients were medically examined between 8 December 2020–6 April 2021, after a median time of 49 days since first positive swab test for SARS-CoV-2. After a median time of 15 months since COVID-19 diagnosis, the same patients were contacted over the phone and investigated by standardized questionnaire collecting information on any persisting symptoms and work ability index (WAI). Four multivariable logistic regression models were fitted to investigate factors associated with persistence of any respiratory, neurological, dysautonomic, or psychiatric symptoms at first (median time 49 days since COVID-19 diagnosis) as well as second (median 15 months since COVID-19 diagnosis) follow up. A multiple linear regression was also employed to investigate factors associated with higher mean WAI, assessed only at second follow up. Additionally, factors associated with persistence of symptoms 200+ days since COVID-19 diagnosis between first and second follow-up were investigated by multivariable Generalized Estimating Equation (GEE). **Results:** At first follow up (median time of 49 days since COVID-19 diagnosis) symptoms more frequently reported were fatigue (80.2%), shortness of breath (69.6%), concentration deficit (44.9%), headache (44.9%), myalgia (44.1%), arthralgia (43.3%), and anosmia (42.1%). At second follow-up (median time of 15 months since COVID-19 diagnosis) 75% patients returned to their baseline status preceding COVID-19. At first follow up males were less likely to experience neurological (OR = 0.16; 95% CI: 0.08; 0.35) as well as psychiatric (OR = 0.43; 95% CI: 0.23; 0.80) symptoms as compared to females. At first follow up, the risk of neurological symptoms increased also linearly with age (OR = 1.04; 95% CI: 1.01; 1.08) and pre-existing depression was a major risk factor for persisting dysautonomic (aOR = 6.35; 95% CI: 2.01; 20.11) as well as psychiatric symptoms (omitted estimate). Consistently, at second follow up only females experience psychiatric symptoms, whereas males exhibited significantly higher mean WAI (RC = 0.50; 95% CI: 0.11; 0.88). Additionally, neurological symptoms at second follow up were more likely in patients with pre-existing comorbidities (OR = 4.31; 95% CI: 1.27; 14.7). Finally, persistence of symptoms lasting 200+ days since COVID-19 diagnosis increased linearly with age (OR = 1.03; 95% CI 1.01–1.05) and were more likely in patients affected by pre-existing depression (OR = 2.68; 95% CI 1.60; 4.49). **Conclusions:** Following a median time of 15 months since first positive swab test, 75% patients with symptoms returned to their baseline health status preceding COVID-19. Females had a significantly lower WAI and were more likely to experience psychiatric symptoms at second follow up (15 months since COVID-19 diagnosis). Furthermore, the risk of symptoms persisting 200+ days since COVID-19 diagnosis increased with history of depression, endorsing the hypothesis that long-COVID-19 symptoms may be at least partially explained by pre-existing psychological conditions. Patient rehabilitation and psychological support may therefore play a key role in caring patients with the so called long COVID-19 syndrome.

## 1. Background

COVID-19 is a systemic disease which can present with a wide clinical spectrum ranging from general respiratory symptoms to multi-organ failure [1,2,3,4], yet in 40% cases patients it may remain asymptomatic [5].

A proportion of patients’ ailments may last after recovering from acute COVID-19 [1,5], with episodic and systemic symptoms of unclear etiology potentially involving the respiratory, cardiovascular, gastrointestinal, neurological, cognitive, musculoskeletal system as well as mental health [5]. Reduced work ability and quality of life have also been reported in relation to persisting COVID-19 symptoms. [6,7,8,9,10].

In October 2021 the World Health Organization (WHO) framed a definition of post COVID-19 as a condition featured by “*history of probable or confirmed SARS-CoV-2 infection, usually three months from the onset of COVID-19, with symptoms that last for at least two months and cannot be explained by an alternative diagnosis*” [11,12]. Syndromic symptoms persisting after acute COVID-19 have been subsequently termed long-COVID-19 by the National Institute for Health and Care Excellence (NICE) of the UK, defining it as a “*collection of signs and symptoms developing during or after an infection consistent with COVID-19, persisting for more than 4 weeks and not explicable by alternative diagnosis*” [13].

A meta-analysis of 15 studies on 47,910 patients aged 17–87 years reported that 80% of patients infected by SARS-CoV-2 developed one or more long-term symptoms (lasting from 14–110 days post-infection), the most common of which being fatigue (58%), headache (44%), attention disorder (27%), hair loss (25%), and dyspnea (24%) [14].

In a more recent comprehensive meta-analysis on 31 studies from 5 July 2021 through 13 March 2022, the pooled global prevalence of post-COVID-19 condition was 0.43 (95% CI: 0.39; 0.46) [15].

Prevalence of persisting COVID-19 symptoms greatly varies between and within countries, ranging from 2.3% [16] to 80% [14], depending on several factors including criteria for follow up time since acute disease, different definitions of long-COVID-19, COVID-19 immune/vaccination status or emergence of new SARS-CoV-2 variants [5]. For instance, the prevalence of long-COVID-19 was estimated to be 5–51% in Italy [17], 1.6–71% in UK [18], 22% in India [19], 35–77% in Germany [7], 49–76% in China [20], 16–53% in USA [21] and 61% in Norway [22].

The risk of long-COVID-19 seems to increase with the severity of acute disease, with higher figures reported among hospitalized patients [6,7,23,24]. In particular, since prevalence long-COVID is estimated to range from 13.3% among non-hospitalized vs. 71% hospitalized patients [6,25,26,27], the latter category has drawn most of the attention thus far for the impact and sequelae of the acute disease. However, there is still no clear evidence on the impact of long-COVID-19 on patients affected by mild/moderate disease [20,28,29,30,31], particularly in Italy, where research has mainly focused on elderly and children populations thus far [32,33].

Understanding persisting symptoms and associated factors is critical to identify patients at risk of developing long-COVID-19 syndrome, taking appropriate interventions to enable patients to return to their normal life, preventing also post COVID-19 disability where possible [20]. Most studies thus far have focused on descriptive collection of post-COVID-19 symptoms on relatively short timelines, focusing on patients recovering from severe disease and hospitalization. Persistence of symptoms >12 months in populations of working age (hence relatively healthier) and their residual ability to work during the initial phase of the pandemic, when more severe SARS-CoV-2 variants strains (as Wuhan and Alpha) were circulating, and most people were still unvaccinated (hence presumably more susceptible) against COVID-19 has not been carried out thus far.

In view of the above, this study aimed to investigate persisting COVID-19 symptoms and associated factors in active workers recovering from confirmed COVID-19 in Trieste (North-eastern Italy), referring to the local post COVID-19 from 8 December 2020 through 6 April 2021—therefore in the early phase of the national vaccination campaign—considering also their residual working ability.

## 2. Methods

A post-COVID-19 clinic was established in Trieste (the main city of Friuli Venezia Giulia Region, North-Eastern Italy) by the University Health Agency Giuliano-Isontina (ASUGI) to support, treat and study patients with COVID-19 sequelae and symptoms persisting after recovering from acute COVID-19. COVID-19 patients investigated in the present study:

- were of working age (i.e., 14–67 years, by Italian law);
- had symptoms persisting for at least three weeks since positive swab test for SARS-CoV-2;
- attended the Long-COVID-19 outpatient clinic of Trieste;
- provided an informed consent to participate to the study.
- Patients were excluded in case of:
- Age < 14 or >67 years
- death during study period;
- inability to sustain a telephone follow-up interview (due to medical reasons as dementia, speech disorders, language barriers, psychiatric disorders, among other);
- refusal to participate to the study;
- loss to follow up.

All patients referring to the post-COVID-19 clinic were examined by a consultant in infectious diseases and additional tests (e.g., laboratory tests and radiological imaging, among other) were performed if need be. A structured self-reported questionnaire was administered to patients to collect information on, date of first positive swab test for SARS-CoV-2, date of symptoms onset, any hospital admission, any symptoms at the start of COVID-19, any symptoms persisting at first follow up (medical examination after median time of 49 days since COVID-19 diagnosis), smoking habit, alcohol consumption and any treatment administered against COVID-19. Patients with symptoms preceding the onset of COVID-19 and shared between COVID-19 and other pre-existing medical conditions were excluded.

At a second follow up (after a median period of 15 months since first positive swab test), study patients were contacted over the phone by qualified doctors to investigate any enduring symptoms using a standardized questionnaire collecting information also on occupation, COVID-19 vaccination status, any re-infections, and any novel health condition emerged.

Additionally, the Work Ability Index (WAI) was also investigated to evaluate the residual work capacity post COVID-19 at second follow-up, stratified as follows:

- 6: No hindrance;
- 5: I am able to do my job, but it causes me some symptoms;
- 4: I must sometimes slow down my work pace or change my work methods;
- 3: I must often slow down my work pace or change my work methods;
- 2: I feel I am able to do only part time work;
- 1: In my opinion I am entirely unable to work.

### 2.1. Outcome Measure

Assessing the persistence of any respiratory, neurological, psychiatric or dysautonomic symptoms (binary outcome: yes vs. no) after a median time of 49 days (first follow up) as well as at 5 months (second follow up) since first positive swab test for SARS-CoV-2. Dysautonomia was defined as a condition including either fatigue, confusion, insomnia, or concentration deficits [34]. Furthermore, the second follow up also included an assessment of the impact of post COVID-19 symptoms on WAI.

### 2.2. Ethical Considerations

The study protocol was approved by the Regional Ethical Committee of Friuli-Venezia Giulia Region (N. 040/2021 approved 7 February 2022) and all participants provided verbal informed consent to participate to the telephone interview.

### 2.3. Statistical Analysis

Categorical variables were expressed as numbers and percentages (%), whereas median (25–75° percentiles) and mean (M) ± standard deviation (SD) were used to describe continuous variables. Comparison of categorical terms was carried out by Chi-Squared test, whereas continuous terms were contrasted by ANOVA test.

COVID-19 symptoms at different time intervals over time were performed by Mc-Nemar (for paired observations) or Mann–Whitney test (for continuous variables).

Factors associated with persistence of any symptoms (respiratory, neurological, psychiatric or dysautonomic, coded Yes vs. No) at <200 as well as 200+ days since initial positive swab test were investigated separately by multivariable logistic regression model, expressing the risk as adjusted odds ratio (aOR) with 95% confidence interval (95% CI). Factors associated with WAI (as linear term) at second follow-up were investigated by multivariable linear regression model, reporting adjusted regression coefficients (aRC) with 95% CI.

Factors associated with persistence of any long-COVID-19 symptoms 200+ days were also investigated by multivariable generalized estimating equation (GEE) models, expressing the results as aOR with 95% CI.

Missing values were excluded from the analysis. A *p*-value < 0.05 was considered significant.

Data were analyzed by STATA version 15 (College Station, TX, USA). Missing data were excluded, and complete case analysis was performed.

## 3. Results

**Figure 1:**
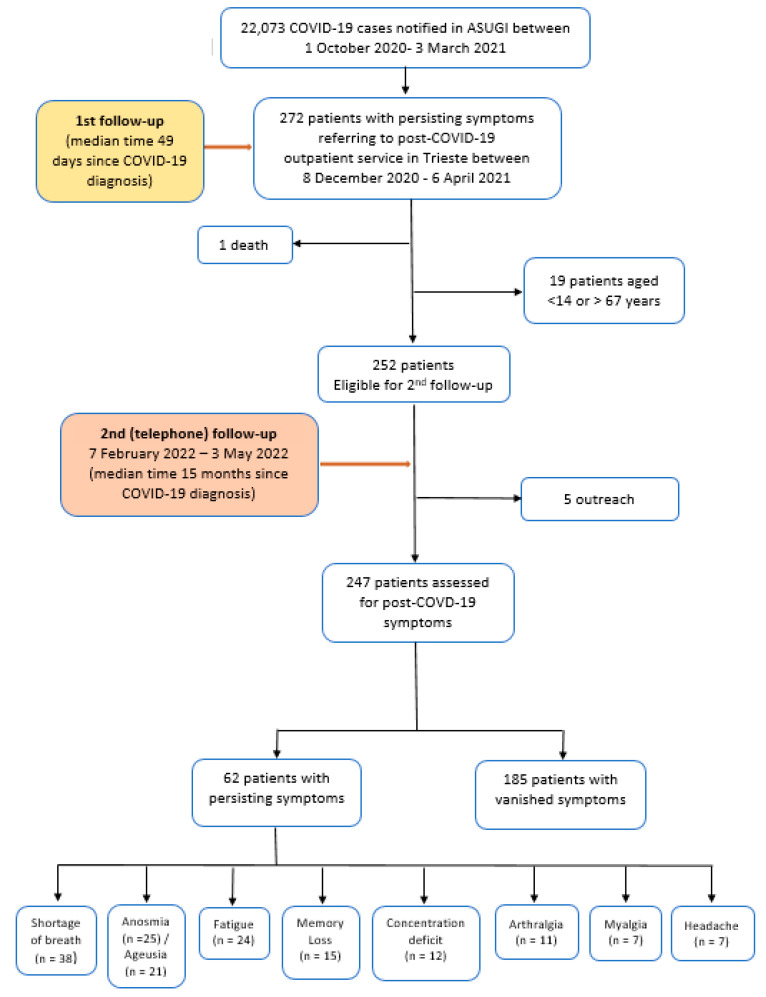
Patients with symptoms assessed at second follow up (median time of 15 months since COVID-19 diagnosis) at post-COVID-19 clinic in Trieste. ASUGI = University Health Agency Giuliano-Isontina.

A total number of 22,073 COVID-19 cases were notified within the catchment area of ASUGI from 1 October 2020 (first diagnosis) through 3 March 2021 (last diagnosis), with 1.2% (=272/22,073) patients referring to the post-COVID-19 outpatient service of Trieste from 8 December 2021 until 6 April 2021. All the latter 272 patients were considered for a second follow up, conducted by telephone interview interview from 7 February 2022 up to 3 May 2022, after a median time of 15 months since COVID-19 diagnosis. Twenty patients were excluded from the study for failing to meet the inclusion criteria (Figure 1). The remaining 252 patients were were interviewed over the phone, five of whom being outreach (Figure 1).

The demographic and clinical characteristics of the study population are shown in Table 1. Two-hundred-forty-seven convalescent patients were investigated over the phone, including 88 (35.6%) males and 159 females. The mean age of the study subjects was 48.1 ± 10.5 years. Twenty-six-point-three percent of (=65/247) patients were smokers, 23.9% (=59/247) had a BMI between 25–29.9 kg/m^2^ and 17.8% (=44/247) > 30 kg/m^2^. Fifty-six percent (=139/247) patients were managed at primary care level. Among 108 hospitalized patients, three were admitted to the intensive care unit.

Concomitant conditions pre-existing SARS-CoV-2 infection were reported in 43.7% (=10/247) patients. The most common comorbidities were thyroid diseases (16.2% = 40/247), allergies (15% = 37/247), and hypertension (14.2% = 35/247). Twenty-seven patients reported depression (10.9% = 27/247). Only one patient had received just one dose of COVID-19 vaccine, 14 days before testing positive for COVID-19 (data not shown). Eighty-seven percent of patients (=215/247) were vaccinated for COVID-19 after being infected by SARS-CoV-2. Twenty percent (=49/247) of patients developed a new condition following COVID-19 and 16.2% (=40/247)

### Post COVID-19 Symptoms

Since the median duration of post COVID-19 symptoms was 200 days, the study population was broken down by two patient groups, those with symptoms lasting <200 days (Group A; *n* = 125) against those with symptoms persisting for 200+ days (Group B; *n* = 122). As can be seen from Table 1, the mean age of Group B patients (49.5 ± 10.2 years) was significantly higher (*p* = 0.002) than Group A (46.8 ± 10.7 years). BMI was also significantly higher (*p* < 0.004) in Group B (27.2 ± 6.2 kg/m^2^) than A (25.2 ± 5.1 kg/m^2^). There was no significant difference in the distribution by sex, smoking status, COVID-19 management, and occupation between the above two patient groups.

As can be noted from Table 1, 49.2% (=122/247) patients reported symptoms lasting 200+ days, and the prevalence of pre-existing depression varied from 16.4% (=20/122) in Group B vs. 5.6% (=7/125) in Group A (*p* = 0.007).

There was no significant difference between the two patient groups in terms of: rate of any re-infections (19.2% in Group A vs. 13.1% in Group B); post-COVID-19 vaccination status (87.2% vs. 86.9%, respectively) and any novel health condition emerging after COVID-19 (17.6% vs. 22.5%, respectively). In particular, six patients were newly diagnosed with post-COVID-19 brain fog, three of whom showing cerebral damage at [18F]-fluorodeoxyglucose positron emission tomography computed tomography scan (^[18F]^-FDG-PET/CT), with areas of hypo metabolism in the parietal and temporal areas of the brain.

As can be noted always from Table 1, the mean WAI was significantly higher (*p* < 0.001) in Group A (WAI = 5.18 ± 1.08) than in Group B (WAI = 4.5 ± 1.44). Moreover, the prevalence of patients experiencing at least some symptoms at work (WAI ≤ 5) was significantly higher (*p* = 0.003) in Group B (67.2% = 82/122) than in Group A (47.2% = 59/125).

**Figure 2:**
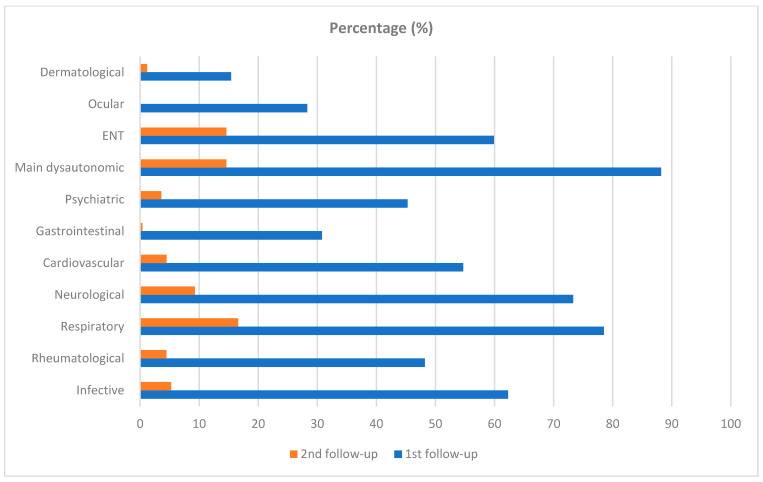
Prevalence (%) of symptoms by follow up (2nd vs. 1st). ENT = Ear-Nose-Throat.

**Figure 3:**
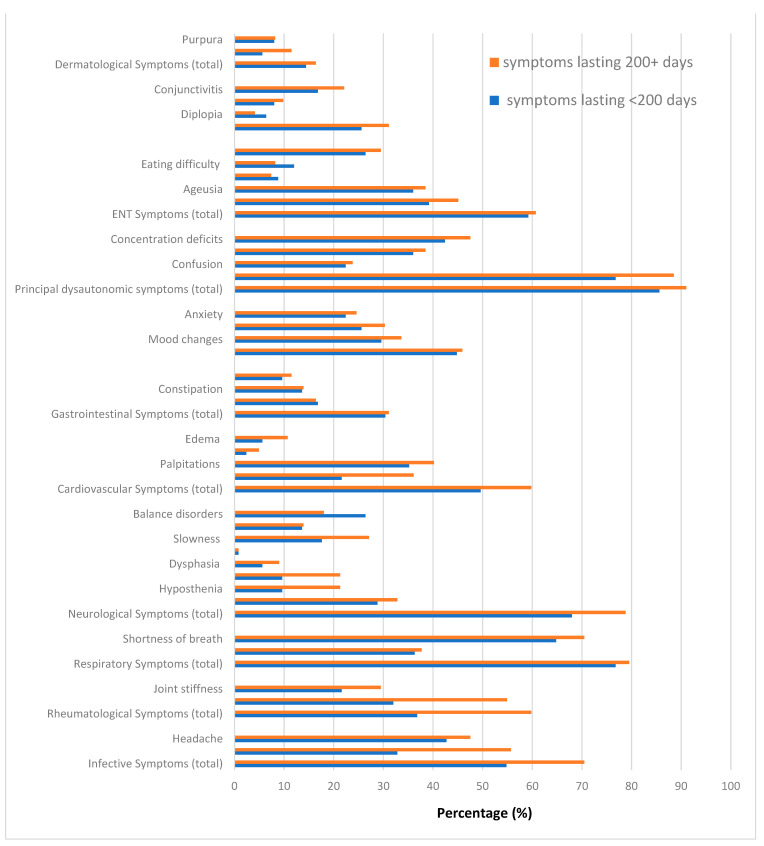
Distribution of symptoms by duration. Group A = patients with symptoms lasting <200 days since COVID-19 diagnosis. Group B = patients with symptoms lasting 200+ days since COVID-19 diagnosis.

**Figure 4:**
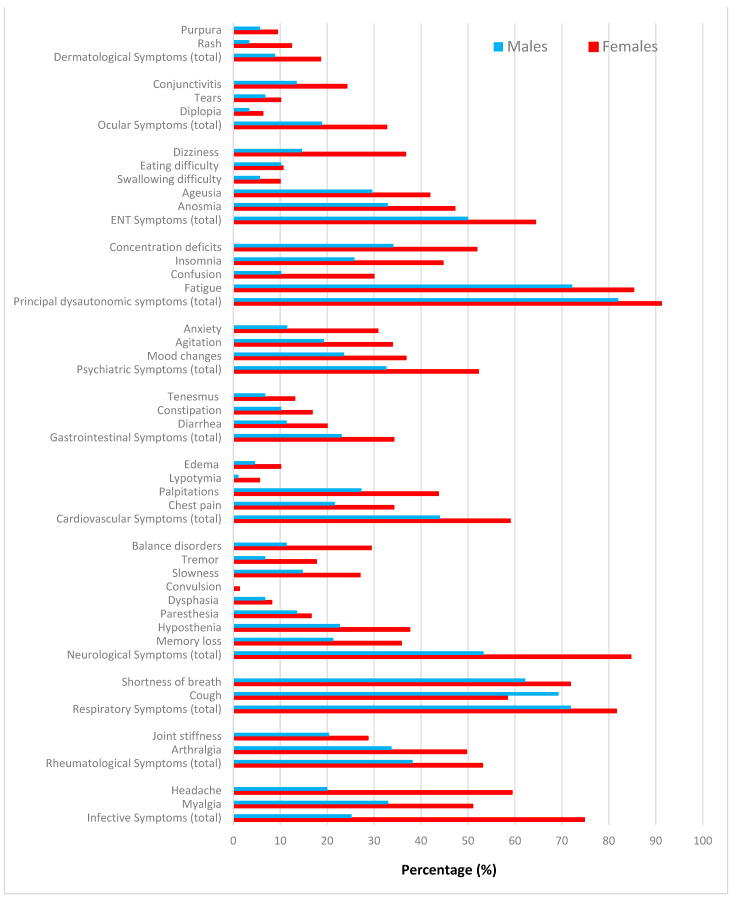
Distribution of any post-COVID-19 symptom (regardless of the duration), by sex of patients.

Figure 2 displays the distribution of symptoms by follow up (2nd vs. 1st). Figure 3 shows the distribution of symptoms by duration (200+ vs. <200 days). Figure 4 displays the distribution of any post-COVID-19 symptom (regardless the duration), by sex of patients.

Table 2 displays the distribution of all symptoms by patient group (A vs. B) as well as by follow up time (2nd vs. 1st). As can be seen, the prevalence of any infective symptoms (70.5% vs. 55.2%; *p* = 0.027), rheumatological symptoms (59.8% vs. 36.8%; *p* < 0.001), paresthesia (21.3% vs. 9.6; *p* = 0.011) and chest pain (37.7% vs. 22.4%; *p* = 0.011) was significantly higher in Group B (patients with symptoms persisting 200+ days since COVID-19 diagnosis).

As can be seen from Table 2, at first follow-up (medical examination)—median time 49 days since first positive swab test—the most common persisting symptoms were dysautonomic (88.2% = 218/247), followed by respiratory (78.5% = 194/247) and neurological (73.3% = 181/247). In particular, the vast majority of patients complained fatigue (80.2% = 198/27)) and shortage of breath (69.6% = 172/247), followed by headache (45% = 111/247), concentration deficits (44.9% = 111/247), myalgia (44.1% = 109/247), arthralgia (43.3% = 107/247), anosmia (42.1% = 104/247) and ageusia (37.2% = 92/247). All subjects affected by pre-existing depression complained dysautonomic symptoms (data not shown).

At second (telephone) follow-up—a median time of 15 months since COVID-19 diagnosis—most patients (74.9% = 185/247) returned to their baseline health status pre-existing COVID-19 against 62 patients with at least one persisting symptoms. The 62 patients with symptoms also at second follow up experienced mainly predominantly shortness of breath (61.3% = 38/62), fatigue (38.7% = 24/62), anosmia (40.3% = 25/62), ageusia (33.9% = 21/62), memory loss (24.1% = 15/62), and concentration deficit (19.4% = 12/62) (Table 2).

Table 3 reports the results of three multiple logistic regression models investigating the risk of the main post-COVID-19 symptoms separately (respiratory, neurological, psychiatric or dysautonomyc) at first as well as second follow up. As can be seen, at the first follow up, males were less likely to experience neurological (aOR = 0.16; 95% CI 0.08; 0.35) as well as psychiatric (aOR = 0.43; 95% CI: 0.23; 0.80) symptoms, whose additional yet major risk factor was depression (aOR= 6.35; 95% CI: 2.01; 20.11). Neurological symptoms increased also linearly with age (aOR = 1.04; 95% CI: 1.01; 1.08). By contrast, dysautonomic symptoms were less likely in males (omitted risk estimate since this sub-group was entirely composed of females) and in patients who had been hospitalized during acute COVID-19 (aOR = 0.31; 95% CI: 0.11; 0.90). The majority of patients with pre-existing depression (73.7% = 14/19) were non-hospitalized during acute COVID-19 (data not shown).

As can be also noted from Table 3, at the second follow up, males were less likely to experience psychiatric symptoms (omitted risk estimate since this sub-group was entirely composed of females) and had also a significantly higher WAI (aRC = 0.50 (0.11; 0.88). Furthermore, the risk of neurological symptoms increased with pre-existing co-morbidities (aOR = 4.31; 95% CI: 1.27; 14.7). Pre-existing co-morbidities were more prevalent in females (53.9% = 11/19) and in patients with pre-existing depression (57.9% = 11/19). Moreover, the hospitalization rate for acute COVID-19 in patients with pre-existing co-morbidities was 49.2% (=30/61)).

Table 4 shows the results of a multivariable GEE investigating persistence of any symptoms 200+ days since initial COVID-19 diagnosis between first and second follow-up. As can be noted, duration of symptoms 200+ days increased linearly with age (aOR = 1.03; 95% CI: 1.0; 1.05) and pre-existing depression syndrome (aOR = 2.68; 95% CI: 1.60; 4.49).

**Figure 5:**
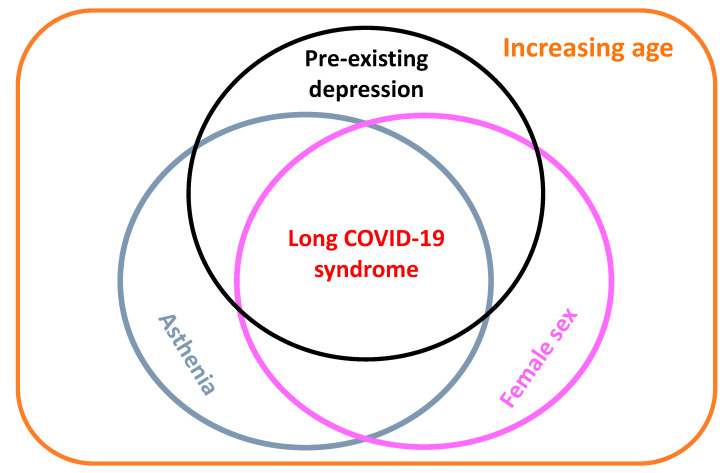
Conceptual framework (created by LC and FLF) explaining the relationships of the main risk factors with post-COVID-19 symptoms.

Figure 5 illustrates the conceptual framework explaining the relationships between the main risk factors and long-COVD-19, according to findings of the present study.

## 4. Discussion

### 4.1. Key Findings

The present study investigated post-COVID-19 symptoms in a group of 247 patients of working age, 43.7% of whom had been admitted to hospital during acute COVID-19 and only three to intensive care unit. Patients referring to the post-COVID-19 clinics of Trieste were followed-up after a median time of 15 months since initial COVID-19 diagnosis. Approximately half study patients had at least one concomitant condition pre-existing COVID-19, most frequently, thyroid disorders, allergies, hypertension, and depression.

At the first follow up (medical examination after a median time of 49 days since first positive swab test), patients complained mainly of dysautonomic (95.5%), ENT (59.9%), respiratory (78.5%), and neurological (73.3%) symptoms. Specifically, the most prevalent symptoms were fatigue (80.2%) and shortage of breath (69.6%).

At second (telephone) follow up, after a median time of 15 months since positive swab test result, 75% patients returned to their baseline health status preceding COVID-19, regardless of hospitalization during the acute disease. The 62 patients still symptomatic at second follow up mainly reported shortness of breath (66.1%), fatigue (38.7%), anosmia (40.3), ageusia (33.9%) and a small proportion (2%) complained of “brain fog”, with symptoms present both at the first as well as the second follow up.

As expected, the mean WAI (measured only at second follow up) was significantly lower in patients reporting any symptoms at 200+ days since COVID-19 diagnosis than in those with symptoms lasting <200 days. Likewise, the prevalence of patients with WAI ≤ 5 was higher in Group B (patients with symptoms persisting 200+ days since COVID-19 diagnosis).

At the first follow up, the risk of neurological, psychiatric and dysautonomic conditions was significantly higher in female patients. Furthermore, at first follow up the risk of neurological symptoms increased linearly with age, whereas dysautonomic symptoms were inversely associated with hospitalization during acute COVID-19.

At the second follow up, females were also more likely to experience psychiatric symptoms and had a significantly lower mean WAI than males. Moreover, pre-existing co-morbidities increased the risk of neurological conditions. Pre-existing comorbidities were more prevalent in females and in patients affected by pre-existing depression, whereas half patients affected by pre-existing comorbidities were hospitalized for acute COVID-19.

The risk of symptoms lasting 200+ days since COVID-19 diagnosis between first and second follow up increased with pre-existing depression and (linearly) with age.

### 4.2. Generalizability

In an online survey conducted from 6 September 2020 through 25 November 2020 on 3762 participants with confirmed COVID-19 diagnosis from 56 different countries, 203 different symptoms in 10 different organs lasting 28+ days were detected, 66 of which persisting over 7 months [33].

Consistently with the open literature, in the present study, the main symptoms experienced by patients at the second follow up were shortage of breath, fatigue, anosmia, and ageusia [7,20,28,30,35,36,37].

The risk of long-COVID-19 reportedly increases with demographic characteristics, comorbidities, immunological response, and severity of the acute disease [4,25,38,39,40]. For instance, in a case control study contrasting 86 patients with long-COVID-19 vs. 39 healthy controls, long COVID-19 symptoms correlated with severity of neuro-immune and neuro-oxidative pathways during the acute phase of the disease, particularly body temperature and release of pro-inflammatory cytokines [41]. Surprisingly the type of care received (primary vs. hospital) during acute COVID-19 did not influence post-COVID-19 symptoms in the present study.

Apart from brain fog, which can be identified at brain FDG PET scan as hypo-metabolism in cortical areas [42,43,44], in the present study, the majority of symptoms were self-reported. Asthenia, a condition difficult to define with wide variability between patients, was the most prevalent persisting symptom in the present study. Asthenia is a common condition accounting for 30% of all consultations in ambulatory settings [45]. Ten percent cases of asthenia are attributable to chronic fatigue syndrome (CFS), and depression is the most common cause of fatigue, accounting for half of cases worldwide. CFS should be considered in case of fatigue, cognitive difficulties, sleep disturbance, and chronic pain, all symptoms frequently observed following COVID-19 [31].

At present, there is still “little scattered of bits of evidence” around the pathogenesis of long COVID-19, which shares some clinical features with the so called “chronic fatigue syndrome”, also known as myalgic encephalomyelitis [38]. Long COVID may supposedly be caused by SARS-CoV-2 direct injury or associated immune/inflammatory response [40] and a recent speculative hypothesis supported the role of inflammatory micro-clots in different organs to explain the multi-systemic symptoms of long-COVID-19 syndrome [37]. In an early study at the beginning of the pandemic, comparing the plasma samples of 13 healthy volunteers, 15 COVID-19 patients, 10 diabetic patients, and 11 patients with long COVID-19 symptoms, clotting was much more prevalent among those with acute as well as long COVID-19 [45]. Likewise, another investigation reportedly found micro-clots in blood samples of all 80 patients affected by long-COVD-19 [46]. Micro-clots, which can also be found in the blood of patients suffering from neurological conditions as Alzheimer or Parkinson disease, are supposed to be triggered by the interaction between the Spike protein of SARS-CoV-2 and fibrinogen and their prevalence increases with severity of acute COVID-19 [38].

At the first follow up, females were by far more likely to experience persisting psychiatric and neurological symptoms following acute COVID-19, a finding in line with the open literature [7,8,33]. Moreover, females also had a significantly lower WAI at the second follow up. An additional risk factor for neurological symptoms at second follow up were pre-existing co-morbidities, which were more prevalent in females and in patients affected by pre-existing depression. According to a recent meta-analysis, female sex and pre-existing asthma were significant risk factors for post-COVID-19 syndrome [47].

The impact of depression on persistence of psychiatric symptoms at first follow up was considerable. Likewise, persistence of COVID-19 symptoms for 200+ days at the second follow up increased with age and pre-existing depression in the present study. Although a potential misclassification bias shall not be ruled out—considering the tight association between dysautonomic symptoms and depression—hospitalization during acute COVID-19 was inversely related with persistence of dysautonomic symptoms, most likely since the majority of patients with pre-existing depression were non-hospitalized.

Depression is a leading cause of disability worldwide, estimated to affect 300 million individuals across all ages [48]. Depression is a condition by far more prevalent among females in the general population [48,49,50,51,52,53,54]. There is some consensus that the persistence of long-COVID-19 symptoms may be at least partially explained by pre-existing psychological conditions (Figure 5) [31]. The restrictive health protection measures (especially isolation and social distancing) enforced during the early phase of the pandemic (when patients of this study were infected and referred to the post-COVID-19 clinical service of Trieste) and the subsequent financial hardships heavily hit the mental health of the general population, worsening any pre-existing psychiatric disorders [31,53,54]. In a systematic review and meta-analysis, risk factors for depression, anxiety, and insomnia during epidemic outbreaks in fact included (among others) female sex, pre-existing physical disorders, psychiatric conditions, and SARS-CoV-2 infection [54].

Following a median time of 15 months since diagnosis, 75% patients returned to their baseline status preceding COVID-19, although 33.2% reported a degree of limitation of their working capacity. In the above online survey on 3762 participants with confirmed COVID-19 diagnosis from 56 countries, 1700 respondents (45.2%) had to reduce their work schedule after SARS-CoV-2 infection, and a further 839 (22.3%) subjects were prevented from working due to post-COVID-19 conditions [33]. Likewise, in a study contrasting 109 post-COVID-19 patients against on 38 controls, those recovering from severe COVID-19 reportedly had poorer performance in tasks requiring high visual sensitivity and were affected by slower processing information speed, as a possible damage to visual or nervous system. By contrast, work performance of patients with mild/moderate COVID-19 was similar to never infected subjects in the latter study [9].

### 4.3. Strengths and Limitations

To the best of our knowledge, this is the first Italian study investigating the persistence of long COVID-19 symptoms more than one year since primary infection. The present study focused on a group of patients of working age (hence on average younger and healthier).

Patients considered for a second follow-up interview were those accessing the post-COVID-19 clinics of Trieste between 8 December 2020 and 6 April 2021, with the national vaccination campaign against COVID-19 in Italy being launched on 27 December 2020. Therefore, all of these patients but one either contracted COVID-19 before the start or during the early phase of the vaccination campaign, when only vulnerable individuals or health care workers could receive the vaccination. Only one study patient was therefore vaccinated for COVID-19 before primary SARS-CoV-2 infection.

Furthermore, although we did not have information on SARS-CoV-2 variants of concern, which may have a variable impact on severity of acute COVID-19 and the respective sequalae, study patients contracted COVID-19 during a period of the pandemic when the original Wuhan and Alpha SARS-CoV-2 variants were predominantly circulating in Italy. This point somehow re-enforces the findings of the present study, as it can reasonably be argued that the impact of long COVID-19 may have been decreasing afterwards, with immunity by vaccination or natural infection progressively spreading across the general population and with the emergence of new variants (as Omicron) reportedly being more infectious yet less pathogenic [55,56].

Considering the tight association between depression and dysautonomic symptoms, a potential selection bias shall not be ruled out, since it is reasonable for a depressed patient to experience insomnia or other mood disorder independently from COVID-19. However, since a follow-up design was employed, we believe pre-existing depression may still play a key effect on dysautonomia. In fact, hospitalization was inversely associated with persistence of dysautonomic symptoms in the present study, since the majority of patients with pre-existing depression were not hospitalized for acute COVID-19.

Nevertheless, the power of this study is relatively limited and refers to a single study center. Furthermore, as with most follow up studies on COVID-19, since most symptoms were self-reported by patients, recall bias should not be neglected. We could not take into account the impact of all relevant confounders, as detailed clinical information on acute disease was not available.

## 5. Conclusions

Following a median time of 15 months since diagnosis, 75% patients with symptoms returned to their baseline health status preceding COVID-19. The main symptoms persisting at 15 months were aspecific and included fatigue, shortage of breath, anosmia and reduced WAI. Females had a significantly lower WAI and were more likely to experience psychiatric as well as neurological symptoms at first follow up. The risk of any long COVID-19 symptoms lasting 200+ days between first and second follow up increased linearly with age and history of depression, endorsing the hypothesis that long COVID-19 syndrome may be at least partially explained by pre-existing psychological conditions. Depression may in fact explain chronic fatigue, by far the most prevalent long-COVID-19 symptom in our study as well as in the open literature.

Although symptoms persisting after recovery from COVID-19 are quite common, their etiology may not be entirely linked to long lasting effects of the viral disease, rather to pre-existing yet predisposing psychological conditions, with bio-psychosocial effects playing a major role. Patient rehabilitation and psychological support should therefore be a key factor to be considered in caring patients suffering from post-COVID-19 symptoms.

## Figures and Tables

**Table 1:** Distribution of patients by duration of any post-COVID-19 symptoms (200+ vs. <200 days). Number (N), column percentage (%), mean (M) ± standard deviation (SD) and *p*-value of ANOVA or chi square test. Significant *p*-values are marked in bold.

				Symptoms Duration N (%)		Total	*p*-Value
				Group A (<200 Days)	Group B (200+ Days)		
**Total patients—N (row %)**				125 (50.6)	122 (49.4)	247 (100)	
**Sex**			**Females**	81 (64.8)	78 (63.9)	159 (64.4)	0.880
			**Males**	44 (35.2)	44 (36.1)	88 (35.6)	
**Age** (years)			**(Mean** **± SD)**	46.8 ± 10.7	49.5 ± 10.2	48.1 ± 10.5	0.020 *
**Smoking**			**Non smokers**	59 (47.2)	69 (56.6)	128 (51.8)	0.319
			**Smokers**	37 (29.6)	28 (22.9)	65 (26.3)	
			**Ex-smokers**	29 (23.2)	25 (20.5)	54 (21.9)	
**BMI (Kg/m^2^)**			**Mean** **± SD**	25.2 ± 5.1	27.2 ± 6.2	26.2 ± 5.7	0.004 *
			**<25**	57 (45.6)	50 (41)	107 (43.3)	0.100
			**25–30**	28 (22.4)	31 (25.4)	59 (23.9)	
			**>30**	15 (12.0)	29	81 (32.8)	
			**Missing**	25 (20)	12 (9.8)	37 (15.0)	
**Pre-existing Comorbidities**			**No**	77 (61.6)	62 (50.8)	139 (56.3)	0.080
			**Yes**	48 (38.4)	60 (49.2)	108 (43.7)	
**Pre-existing** **Comorbidities**			**Hypertension**	15 (12)	20 (16.4)	35 (14.2)	0.322
			**Diabetes**	2 (1.6)	6 (4.9)	8 (3.2)	0.141
			**Cardiovascular Disease**	6 (4.8)	7 (5.7)	13 (5.3)	0.741
			**Thyroid Disease**	19 (15.2)	21 (17.2)	40 (16.2)	0.668
			**Asthma**	7 (5.6)	9 (7.4)	16 (6.5)	0.571
			**COPD**	6 (4.8)	7 (5.7)	13 (5.3)	0.741
			**Cancer**	3 (2.4)	2 (1.6)	5 (2.0)	0.671
			**Allergy**	18 (14.4)	19 (15.6)	37 (15.0)	0.796
**Depression**			**No**	118 (94.4)	102 (83.6)	193 (78.1)	**0.007**
			**Yes**	7 (5.6)	20 (16.4)	27 (10.9)	
**COVID-19 care**	**Primary**			70 (56)	69 (56.56)	139 (56.3)	0.990
	**Hospital**		**Total**	55 (44)	53 (43.44)	108 (43.7)	0.990
			**Non-Intensive Care**	53 (42.4)	52 (42.64)	105 (42.5)	0.930
			**Intensive Care**	2 (1.6)	1 (0.8)	3 (1.2)	0.576
**Occupation**	**Blue Collar workers**			15 (12)	14 (11.5)	29 (11.7)	0.514
	**White Collar workers**			38 (30.4)	32 (26.2)	70 (28.3)	
	**Health Care workers**			29 (23.2)	36 (29.5)	65 (26.3)	
	**Trade and transport workers**			11 (8.8)	14 (11.5)	25 (10.1)	
	**Law enforcement’s**			5 (4)	7 (5.7)	12 (4.9)	
	**Education workers**			22 (17.6)	12 (9.8)	34 (13.8)	
	**Other (unemployed, housewives, retirees)**			5 (4)	7 (5.7)	12 (4.9)	
**Re-infection**			**No**	101 (80.8)	106 (86.9)	207 (83.8)	0.194
			**Yes**	24 (19.2)	16 (13.1)	40 (16.2)	
**Post-COVID-19** **Vaccination**			**No**	16 (12.8)	16 (13.1)	32 (13.0)	0.941
			**Yes**	109 (87.2)	106 (86.9)	215 (87.0)	
**Novel condition** **developed after COVID-19**			**No**	103 (82.4)	93 (77.5)	196 (79.4)	0.418
			**Yes**	22 (17.6)	27 (22.5)	49 (19.8)	
**Work Ability Index (WAI)**		**Mean** **± SD**		5.18 ± 1.08	4.5 ± 1.44	4.9 ± 1.3	<0.001 *
		**No obstacles to work (6)**		66 (52.8)	39 (32.2)	105 (42.7)	**0.003**
		**Some symptoms (5)**		31 (24.8)	33 (27.3)	64 (26.0)	
		**Occasionally slowing down (4)**		17 (13.6)	18 (14.9)	35 (14.2)	
		**Frequently slowing down (3)**		8 (6.4)	22 (18.2)	30 (12.2)	
		**Only part time work (2)**		2 (1.6)	2 (1.6)	4 (1.2)	
		**Unable to work at all (1)**		1 (0.8)	7 (5.8)	8 (3.2)	

* ANOVA test.

**Table 2:** Distribution of persisting symptoms in 247 COVID-19 patients, by duration (200+ vs. <200 days) and follow up timeline (FU, 2nd vs. 1st). * Chi-square test; ** Mc-Nemar test. ENT = Ear-Nose-Throat.

Symptoms	Duration N (Col %)		*p*-Value *	Post-COVID-19 FU N (Col %)		*p*-Value **
	(<200 Days)	(200+ Days)		1st	2nd	
**Total Patients**	**125 (50.6)**	**122 (49.4)**		**247 (100)**	**247 (100)**	
**With persisting symptoms**	**125 (100)**	**122 (100)**		**247 (100)**	**62 (25.1)**	<0.001

**Infective Symptoms (total)**	**68 (55.2)**	**86 (70.5)**	**0.014**	**155 (62.3)**	**13 (5.3)**	**<0.001**
Myalgia ^§^	41 (32.8)	68 (55.7)	<0.001	109 (44.1)	7 (2.8)	<0.001
Headache ^§^	53 (42.7)	58 (47.5)	0.404	111 (44.9)	7 (2.8)	<0.001

**Rheumatological Symptoms (total)**	**46 (36.8)**	**73 (59.8)**	**<0.001**	**119 (48.2)**	**11 (4.5)**	**<0.001**
Arthralgia	40 (32.0)	67 (54.9)	<0.001	107 (43.3)	11 (4.5)	<0.001
Joint stiffness	27 (21.6)	36 (29.5)	0.127	63 (25.5)	3 (1.2)	<0.001

**Respiratory Symptoms (total)**	**96 (60.8)**	**97 (79.5)**	**0.784**	**194 (78.5)**	**41 (16.6)**	**0.210**
Cough	45 (36.3)	49 (40.2)	0.636	91 (36.8)	4 (1.6)	<0.001
Shortness of breath ^§^	81 (64.8)	92(75.4)	0.226	172 (69.6)	38 (15.3)	<0.001

**Neurological Symptoms (total)**	**85 (68.0)**	**96 (78.7)**	**0.092**	**181 (73.3)**	**23 (9.3)**	**<0.001**
Memory loss ^§^	36 (28.8)	40 (32.8)	0.525	76 (30.8)	15 (6.1)	<0.001
Hyposthenia ^§^	33 (26.4)	46 (37.7)	0.056	79 (32.0)	7 (2.8)	<0.001
Paresthesia ^§^	12 (9.6)	26 (21.3)	0.011	38 (15.4)	3 (1.2)	<0.001
Dysphasia	7 (5.6)	11 (9.0)	0.225	18 (7.3)	3 (1.2)	<0.001
Convulsion	1 (0.8)	1 (0.8)	0.991	2 (1.0)	1 (0.4)	<0.001
Slowness	22 (17.6)	33 (27.1)	0.063	55 (22.3)	1 (0.4)	<0.001
Tremor	17 (13.6)	17 (13.9)	0.919	34 (13.8)	2 (0.8)	<0.001
Balance disorders	33 (26.4)	22 (18.0)	0.114	55 (22.3)	0	<0.001

**Cardiovascular Symptoms (total)**	**62 (49.6)**	**73 (59.8)**	**0.210**	**135 (54.7)**	**11 (4.5)**	**<0.001**
Chest pain ^§^	28 (22.4)	46(37.7)	0.011	72 (29.1)	2 (0.8)	<0.001
Palpitations ^§^	44 (35.2)	50 (41.0)	0.376	93 (37.7)	9 (3.6)	<0.001
Lipothymia ^§^	3 (2.4)	7 (5.7)	0.188	9 (3.6)	0	<0.001
Edema	7 (5.6)	13 (10.7)	0.145	20 (8.1)	0	<0.001

**Gastrointestinal Symptoms**	**38 (30.4)**	**38 (31.1)**	**0.934**	**76 (30.8)**	**1 (0.4)**	**<0.001**
Diarrhea ^§^	21 (16.8)	21 (16.4)	0.954	41 (16.6)	1 (0.4)	<0.001
Constipation ^§^	17 (13.6)	17 (13.9)	0.939	34 (13.8)	0	<0.001
Tenesmus	12 (9.6)	14 (11.5)	0.631	26 (10.5)	0	<0.001
**Psychiatric Symptoms (total)**	**56 (44.8)**	**56 (45.9)**	**0.908**	**112 (45.3)**	**9 (3.6)**	**0.385**
Mood changes	37 (29.6)	43 (35.2)	0.391	78 (31.6)	9 (3.6)	<0.001
Agitation	32 (25.6)	40 (32.9)	0.264	69 (27.9)	0	<0.001
Anxiety	28 (22.4)	31 (25.4)	0.654	58 (23.5)	2 (0.8)	<0.001

**Main dysautonomic symptoms**	**107 (85.6)**	**111 (91.0)**	**0.262**	**218 (88.2)**	**36 (14.6)**	**<0.001**
Fatigue ^§^	96 (76.8)	108 (88.5)	0.127	198 (80.2)	24 (9.7)	0.003
Confusion	28 (22.4)	29 (23.8)	0.826	57 (23.1)	4 (1.6)	<0.001
Insomnia ^§^	45 (36.0)	47 (38.5)	0.515	92 (37.2)	5 (2.0)	<0.001
Concentration deficits	53 (42.4)	58 (47.5)	0.404	111 (44.9)	12 (4.8)	<0.001
Fatigue & Concentration deficit ^§^	45 (36.0)	55 (45.1)	0.176	101 (40.9)	4 (2.0)	<0.001

**ENT Symptoms (total)**	**74 (59.2)**	**74 (60.7)**	**0.877**	**148 (59.9)**	**36 (14.6)**	**<0.001**
Anosmia	49 (39.2)	55 (45.1)	0.349	104 (42.1)	25 (10.1)	<0.001
Ageusia	45 (36.0)	47 (38.5)	0.682	92 (37.2)	21 (8.5)	<0.001
Swallowing difficulty ^§^	11 (8.8)	9 (7.4)	0.682	20 (8.1)	3 (1.2)	<0.001
Eating difficulty ^§^	15 (12.0)	10 (8.2)	0.322	25 (10.1)	0	<0.001
Dizziness ^§^	33 (26.4)	36 (29.5)	0.586	69 (27.9)	7 (2.8)	<0.001

**Ocular Symptoms (total)**	**32 (25.6)**	**38 (31.1)**	**0.423**	**70 (28.3)**	**0**	**<0.001**
Diplopia	8 (6.4)	5 (4.1)	0.418	13 (5.3)	0	<0.001
Tears	10 (8.0)	12 (9.8)	0.613	22 (8.9)	0	<0.001
Conjunctivitis	21 (16.8)	27 (22.1)	0.158	48 (19.4)	0	<0.001

**Dermatological Symptoms (total)**	**18 (14.4)**	**20 (16.4)**	**0.745**	**38 (15.4)**	**3 (4.8)**	**<0.001**
Rash ^§^	7 (5.6)	14 (1.1)	0.098	21 (8.5)	3 (4.8)	<0.001
Purpura	10 (8.0)	10 (8.2)	0.955	20 (8.1)	0	<0.001

^§^ dysautonomic symptoms according to Blitshteyn et al., 2022 [34].

**Table 3:** Persistence of post-COVID-19 symptoms (respiratory, neurological, psychiatric, dysautonomic, and WAI) at 1st and 2nd follow-up since first positive swab test against SARS-CoV-2. Multiple logistic regression investigating respiratory, neurological, psychiatric, and dysautonomic symptoms (binary outcome: yes vs. no). Multiple linear regression analysis investigating Work Ability Index (WAI, linear term) at second follow up. Adjusted Odds Ratios (aOR, for the multiple logistic regression models) and regression coefficients (aRC, for the multiple linear regression model) with 95% Confidence Intervals (95% CI). Each multivariable regression model at 1st follow up or second follow up was fitted on 210 complete case (analysis) observations. Significant estimates were marked in bold.

Factors		Post-COVID-19 Symptoms				
		Respiratory aOR (95% CI)	Neurological aOR (95% CI)	Psychiatric aOR (95% CI)	Dysautonomic aOR (95% CI)	WAI aRC (95% CI)
**1st follow up (median time: 49 days since COVID-19 diagnosis)**						
	**Female**	Reference	Reference	Reference	Reference	NA
	**Male**	0.52 (0.25; 1.07)	**0.16 (0.08; 0.35)**	**0.43 (0.23; 0.80)**	0.41 (0.19; 1.28)	
**Age (years, linear term)**		0.98 (0.95; 1.02)	**1.04 (1.01; 1.08)**	1.00 (0.98; 1.03)	0.98 (0.93; 1.02)	
**BMI (Kg/m^2^, linear term)**		1.04 (0.97; 1.12)	1.01 (0.94; 1.08)	0.98 (0.93; 1.03)	1.02 (0.93; 1.12)	
**Smoking**	**No**	Reference	Reference	Reference	Reference	
	**Yes**	0.85 (0.41; 1.75)	1.2 (0.59; 2.48)	1.85 (0.03; 3.34)	0.46 (0.17; 1.21)	
Co-Morbidities *	**No**	Reference	Reference	Reference	Reference	
	**Yes**	1.10 (0.52; 2.33)	0.86 (0.41; 1.82)	1.03 (0.55; 1.90)	1.53 (0.56; 4.2)	
**Depression**	**No**	Reference	Reference	Reference	Reference	
	**Yes**	0.78 (0.26; 2.34)	1.47 (0.42–5.20)	**6.35 (2.01; 20.11)**	**Omitted**	
**COVID-19 care**	**Primary**	Reference	Reference	Reference	Reference	
	**Hospital**	0.88 (0.42; 1.83)	0.69 (0.33; 1.43)	0.81 (0.44; 1.49)	**0.33 (0.11; 0.90)**	
**2nd follow up (median time: 15 months since COVID-19 diagnosis)**						
**Sex**	**Female**	Reference	Reference	Reference	Reference	Reference
	**Male**	1.34 (0.61; 2.89)	0.39 (0.11; 1.36)	omitted	0.43 (0.16; 1.16)	**0.50 (0.11; 0.88)**
**Age (years, linear term)**		1.01 (0.97; 1.04)	1.02 (0.96; 1.08)	0.94 (0.86; 1.02)	0.99 (0.95; 1.03)	−0.001 (−0.18; 0.17)
**BMI (kg/m^2^, linear term)**		0.97 (0.90; 1.05)	1.08 (0.997; 1.16)	1.07 (0.96; 1.20)	1.06 (0.99; 1.12)	−0.01 (−0.04; 0.23)
**Smoking**	**No**	Reference	Reference	Reference	Reference	Reference
	**Yes**	0.72 (0.34; 1.53)	0.59 (0.21; 1.43)	0.98 (0.19; 5.18)	0.91 (0.40; 2.07)	0.10 (−0.35; 0.38)
Co-Morbidities *	**No**	Reference	Reference	Reference	Reference	Reference
	**Yes**	1.10 (0.50; 2.39)	**4.31 (1.27; 14.7)**	3.14 (0.46; 21.2)	1.93 (0.40; 2.07)	−0.27 (−0.66; 0.11)
**Depression**	**No**	Reference	Reference	Reference	Reference	Reference
	**Yes**	0.19 (0.02; 1.47)	0.43 (0.08; 2.35)	1.04 (0.13; 8.13)	1.13 (0.35; 3.77)	−0.06 (−0.63; 0.51)
**COVID-19 care**	**Primary**	Reference	Reference	Reference	Reference	Reference
	**Hospital**	0.95 (0.45; 2.03)	0.57 (0.20; 1.62)	0.39 (0.07; 2.27)	1.24 (0.54; 2.84)	0.29 (−0.08; 0.67)

* Including either hypertension, allergy, thyroid disease, cardio-vascular disease, COPD, asthma, or cancer.

**Table 4:** Generalized estimated equation investigating persistence of symptoms (binary endpoint: 200+ vs. <200 days) between 2nd and 1st follow-up. Adjusted odds ratios (aOR) with 95% confidence intervals (95% CI) and stratum specific *p*-values. Significant estimates are reported in bold. BMI = body mass index. Multivariable model fitted on 444 complete case (analysis) observations.

Factors		aOR (95% CI)	*p*-Value
**Sex**	**Females**	reference	0.675
	**Males**	0.97 (0.65; 1.44)	
**Age** (years, linear)		**1.03 (1.01; 1.05)**	**0.001**
**BMI** (kg/m^2^, linear)		1.03 (1.00; 1.06)	0.068
**Depression**	**No**	reference	**0.001**
	**Yes**	**2.68 (1.60; 4.49)**	

## Data Availability

Data generated and analyzed during the current study are not publicly available, since they were purposively collected by the authors for the present study, but are available from the corresponding author on reasonable request.
